# Comparison of Magnetic Resonance Imaging Scales for Assessment of Interval Changes of Arthropathy in Boys with Severe Hemophilia

**DOI:** 10.3390/jcm14134792

**Published:** 2025-07-07

**Authors:** Ningning Zhang, Manuel Carcao, Danial M. Ignas, Brian M. Feldman, Pamela Hilliard, Rahim Moineddin, Ann Marie Stain, Paul Babyn, Victor S. Blanchette, Andrea S. Doria

**Affiliations:** 1 Department of Radiology, Beijing Children’s Hospital, Capital Medical University, National Center for Children’s Health, Beijing 100045, China; 2 Department of Paediatrics, Division of Haematology/Oncology, University of Toronto, The Hospital for Sick Children, Toronto, ON M5G 1X8, Canada; 3 Child Health Evaluative Sciences, The Hospital for Sick Childre, Toronto, ON M5G 0A4, Canada; 4 Division of Rheumatology, The Hospital for Sick Children, Toronto, ON M5G 0A4, Canada; 5 Institute of Medical Science, University of Toronto, Toronto, ON M5S 1A8, Canada; 6 Dalla Lana School of Public Health, University of Toronto, Toronto, ON M5S 3M2, Canada; 7 Department of Rehabilitation, The Hospital for Sick Children, Toronto, ON M5G 1X8, Canada; 8 Department of Family & Community Medicine, University of Toronto, Toronto, ON M5S 3M2, Canada; rahim.moineddin@utoronto.ca; 9 Department of Nursing, The Hospital for Sick Children, Toronto, ON M5G 1X8, Canada; 10 Department of Medical Imaging, University of Saskatchewan, Saskatoon, SK S7N 5A5, Canada; paul.babyn@gmail.com; 11 Department of Diagnostic & Interventional Radiology, The Hospital for Sick Children, University of Toronto, Toronto, ON M5G 0A4, Canada; 12 Department of Medical Imaging, University of Toronto, Toronto, ON M5G 0A4, Canada

**Keywords:** children, hemophilia, arthropathy, magnetic resonance imaging (MRI), prophylaxis

## Abstract

**Background/Objectives:** The variety of magnetic resonance imaging (MRI) scales available to measure soft tissue and osteochondral changes in joints of persons with hemophilia poses challenges in evaluating published clinical/research studies. To evaluate the value of four MRI scales [(i) the 17-point International Prophylaxis Study Group [IPSG] additive scale; (ii) and (iii) the compatible IPSG progressive (P) and additive (A) scales; and (iv) the Denver progressive scale] to assess joint change in boys with hemophilia participating in a prospective two-year prophylaxis study. **Methods:** Boys with severe hemophilia A (ages, 7–16 years) followed at the Hospital for Sick Children, Toronto, Canada had MRI evaluations of six index joints (ankles, knees, elbows) at study entry and exit. Musculoskeletal (MSK) outcomes included in the study were the Colorado Child Physical Examination (PE) scale; the Pettersson (X-ray) scale; and the aforementioned 4 MRI scales. **Results:** Very strong (r ≥ 0.80) correlations were observed between the IPSG 17-point, the IPSG progressive (P) and the Denver MRI scales, and moderate (r = 0.40–0.59) to strong (r = 0.60–0.79) correlations for the IPSG 17 point and the IPSG additive (A) MRI scales. Very weak (r = 0.20–0.39) or no correlations were observed between soft tissue MRI scores and the swelling item of the Child PE scale. **Conclusions:** All four MRI scales demonstrated relative comparability of their construct validities for assessing mild/moderate hemophilic arthropathy. The 17-point IPSG additive scale is recommended as a reference standard in future long-term studies of young boys with hemophilia receiving factor and non-factor-based preventive therapies.

## 1. Introduction

A major complication in persons with hemophilia (PwH) is recurrent bleeding into joints leading to painful and potentially disabling arthropathy. The joints most often affected are the ankles, knees and elbows, often referred to as “index joints”. This undesirable complication of hemophilia can be greatly reduced, and potentially prevented, by preventive therapies including prophylaxis with clotting factor concentrates or non-factor hemostatic agents started at a very young age [1,2,3,4]. However, life-long prophylaxis is extremely expensive, and health care providers involved with the assessment and care of PwH are increasingly required to justify the high cost of preventive therapies by collecting and reporting objective data about joint health [5,6].

Magnetic resonance (MR) imaging is considered the reference standard for the assessment of soft tissue (effusion/hemarthrosis, synovial hypertrophy and hemosiderin) and osteochondral changes (cartilage loss, surface erosions and subchondral cysts) in joints of PwH and bleed-related arthropathy.

The large variety of MR imaging scales available to measure joint changes in hemophilic arthropathy poses challenges for clinicians to evaluate and compare published studies. This manuscript aims to clarify the effectiveness of four different magnetic resonance imaging (MRI) scales previously used in clinical trials of hemophilia to measure soft tissue and osteochondral tissue changes in boys with mild/moderate hemophilic arthropathy. We compared their designed measures (i.e., their abilities to measure what they propose to measure, i.e., construct validity) [7,8] with those of two other non-MRI scales, a standardized joint assessment score based on physical examination (the Colorado Child Physical Examination [PE] scale) [9,10] and a radiographic score for assessment of osteochondral joint changes (the Pettersson scale) [11]. In this two-year prospective randomized prophylaxis study, boys with early hemophilia-related joint disease were enrolled in the study using two different approaches to prophylaxis: a tailored approach adjusted to reported bleeding and a standard non-tailored weight-based approach. Two of these MRI scales are progressive [the Denver MRI [12] and the International Prophylaxis Study Group (IPSG) compatible MRI-P [13] and two are additive [the IPSG compatible MRI-A [13] and the single 17-point IPSG MRI scale [14]. Whereas in progressive scales the numeric score increases as MRI findings progress to categories of abnormalities that are given greater weight, in additive scales equal weight is placed upon each MRI finding and the values of abnormal findings are summed for the total score [6].

## 2. Materials and Methods

### 2.1. Patient Data

The MR images that form the basis of this study were obtained as part of a prospective, two-year, single-center, randomized, prophylaxis study. Enrolled participants were boys with the following characteristics: a diagnosis of severe hemophilia A [circulating factor VIII (FVIII) level < 1%]; ages between 7 and 16 years; no current or past (within two years of study enrolment) history of an inhibitor to FVIII (defined as a neutralizing alloantibody titer of >0.5 Bethesda Units measured using the Nijmegen modification of the Bethesda assay); and demonstrated absence of target joint bleeding defined as ≥3 bleeds into a single index joint (ankle, knee, or elbow) in the 6 months before study enrollment [10]. Eligible subjects were randomized to receive standard, weight-based prophylaxis (SP) [30 IU/kg of a recombinant factor VIII (rFVIII) concentrate on 3 non-consecutive days each week] or tailored prophylaxis (TP) starting at 25 IU/kg on two non-consecutive days each week with dose-escalation up to 40 IU/kg of rFVIII on alternate days based on bleeding into the index joints [15,16,17].

### 2.2. Patient Joint Examinations

All participants enrolled in the randomized prophylaxis study underwent physical examinations, plain radiography (X-ray) and MRI examinations of the six index joints at the study entry. Physical examinations of the index joints were performed every 6 months during the two-year study, and X-ray and MRI examinations were performed at the study exit. The physical examination joint scores and X-ray scores used to evaluate the status of joints, at the time of the study, were considered standard outcome measures for use in clinical trials of prophylaxis [9,10,11].

### 2.3. Physical Examination

The Colorado Child Physical Examination (Child PE) instrument, developed by the Mountain States Regional Hemophilia and Thrombosis Centre to detect early joint changes in boys with hemophilia, was used for this study [9,10] [Supplementary Table S1]. All study joint assessments were completed by one experienced hemophilia clinic physiotherapist (PH).

### 2.4. Radiographic (Plain X-Ray) Scoring System

The Pettersson Score was used for the interpretation of X-rays of the index joints [11] [Supplementary Table S2]. Total scores range from 0 (normal) to 13 (most advanced changes) for each of the index joints (ankles, knees and elbows). The range of scores for the six index joints is 0–78 [11].

### 2.5. MRI Scoring Systems

MR images were scored using the following four MRI scales:

1. 10-point Denver progressive scale [Supplementary Table S3] [12].
2. 10-point Compatible IPSG MRI progressive scale (MRI-P) [Supplementary Table S4] [13].
3. 20-point Compatible IPSG MRI additive scale (MRI-A) [Supplementary Table S4] [13].
4. 17-point IPSG MRI additive scale [Supplementary Table S5] [14].

Details for each of these MRI scoring systems are summarized in the Supplementary Material.

### 2.6. X-Ray Imaging Acquisition

Anterior–posterior lateral view radiographs of the six index joints were obtained at study entry if such images had not been obtained six months before enrollment.

### 2.7. MR Imaging Acquisition

MRI examinations were performed using a 1.5 Tesla General Electric Signa system (Milwaukee, WI, USA) at study entry and exit. Ankles, knees and elbows were imaged using head, cardiac and long extremity (USCTLMID) coils, respectively. The protocol included coronal and sagittal images of the six index joints with additional axial images of elbows using a multiplanar gradient recalled (MPGR) technique [repetition time (TR) 450–667 ms, echo time (TE) 20 ms, flip angle 20°, slice thickness 5 mm, slice spacing 5 mm, field of view (FOV) range 20–30 cm, matrix 256 × 192, number of excitations (NEX) acquisition 2]. The total scan time for six joints varied from 70 to 90 min.

### 2.8. MR Image Interpretation

X-ray and MRI examinations were reviewed by two pediatric radiologists with three and ten years of experience after training at the time of interpretation of the images (N.Z. and A.S.D., respectively), who were blinded to clinical data. Scoring discrepancies of pathologic findings were adjudicated by the more experienced reader (A.S.D.).

### 2.9. Statistical Methods

Descriptive analysis of the available data is reported as medians and ranges. Improvement in scores was reported as −1, −2, −3, where −3 had the most improvement in findings stability as 0, and worsening as +1, +2, +3, where +3 had the most disease progression.

Spearman’s rank correlation coefficients were used to determine the association between the total scores of the MRI scales at study entry and exit. This method was also used to determine the association between the soft tissue scores and various soft tissue components of the MRI and Child PE scales.

Intraclass correlation coefficients (ICCs) and 95% confidence intervals (CIs) were used to assess inter-reader reliability for X-ray and MRI scores. Spearman’s rank correlation coefficients and ICCs ≤ 0.19 indicated very weak, 0.20–0.39 weak, 0.40–0.59 moderate, 0.60–0.79 strong and 0.80–1.0 very strong correlations between the elements being compared [18].

A Kruskal–Wallis test with Bonferroni-adjusted *p*-values (threshold, *p* < 0.008 at the adjusted level) being considered statistically significant was used to compare the IPSG 17-point scoring totals of the ankles, knees and elbows with other scales within the TP and SP groups and between both groups [19,20]). Mann–Whitney U tests with Bonferroni-adjusted *p*-values were used to determine the degree of difference between joint totals in the comparisons that showed significant *p*-values from the Kruskal–Wallis test. Otherwise, for assessment of differences in the frequency of joints with MRI abnormalities between the SP and TP groups, *p* < 0.05 was considered to be statistically significant.

## 3. Results

Twenty-one boys were enrolled. All had baseline physical examinations, X-rays and MRI studies of all six index joints. However, one boy dropped out of the study five months after enrollment, and three participants were unable to complete their imaging assessments due to distance/scheduling issues or inability to undergo MRI examinations due to age. Consequently, complete information on enrollment and end-of-study physical examinations, X-rays and MRI scores were available for 17 of the 21 (80.9%) boys. Of these, eight boys (47.1%) were in the SP group and nine boys (52.9%) were in the TP study arms [Table 1]. These 17 participants constitute the study cohort for this study.

### 3.1. Imaging Studies at Study Entry

Characteristics of the study group at enrollment are detailed in Table 1. As a total study cohort, there were no clinically significant differences between boys randomized to receive SP versus TP, concerning total index joint physical examination and plain radiograph (Pettersson) scores.

The proportion of joints at study entry with any abnormal soft tissue or osteochondral findings using the 17-point IPSG MRI scale is detailed in Table 1. Although numerically at study entry the joints of the TP group showed higher 17-point IPSG MRI soft tissue total scores representing more pathology as compared to the SP participants [median score (range): 6 (0–15) and 2.5 (0–11), respectively, (*p* = 0.81)] and osteochondral total scores [5 (0–10) and 0 (0–4)] (*p* = 0.32); no statistically significant differences were noted between the two groups.

Although at entry MRI examinations 35/54 (64.8%) of the joints of patients in the TP cohort had zero soft tissue scores compared to 36/48 (75.0%) in the SP cohort, concerning osteochondral changes more joints of patients in the TP cohort presented with pathology (20.4%) than joints of patients in the SP group (6.3%) (*p* = 0.047). Further, there was a tendency towards a difference in the total scores between the two groups at exit MRI examinations, with more joints of patients in the TP cohort presenting with pathologic changes (42.6%) than those of patients in the SP cohort (23.4%) (*p* = 0.057) [Table 2].

### 3.2. Changes in Joint Scores over the Study Period

Of all the 101 index joints evaluated in 17 patients who underwent MR imaging at study entry and exit, only 16/101 (15.8%) joints (13 ankles, 2 elbows, and 1 knee) showed an observable change in 17-point IPSG MRI scores. Of those, 10 ankles and 1 elbow (10.9%) demonstrated score worsening using this MRI scale [Table 3 and Table S6]. In the majority of cases, index joints did not show any observable changes over the two-year study period.

Of the joints that deteriorated, nine (75%) were from participants from the TP group, while three (25%) were from the SP group. Four out of seven (57.1%) participants of the TP arm and one out of three (33.3%) of the SP arm presented with interval deterioration of osteochondral structures of their joints. Improvement in total joint scores (−1 to −2) occurred in six joints (five ankles and one knee) of four patients. Of the joints that improved, three (50%) belonged to three patients who were on TP and three (50%) from one patient of the SP cohort.

Overall, most changes in scores from study entry to exit were observed in ankles, followed by elbows, with scores of ±1 most frequently observed [Table 3]. Overall, soft tissue changes were more commonly observed at study entry than osteochondral changes [Table 3].

### 3.3. Tailored Prophylaxis Group [Nine Boys (54 Index Joints) Who Completed MRI Evaluations Were Available at Study Entry and Exit]

The majority of index joints (44/54, 81.5%) did not show any changes over the study period. A change in MRI scores between study entry and exit of at least 1 point was observed in 10/54 (18.5%) index joints (eight ankles and two elbows) in seven of nine (77.8%) boys [Table 3]. Total joint scores improved in two index joints (both ankles) in two boys. In both of these joints, the improvement was noted in the category of effusion/hemarthrosis. As expected, there were no improvements in osteochondral subscores. Total joint scores worsened in eight index joints of seven boys. In six index joints (five ankles and one elbow), there was a deterioration of only (+1) point score. Participant 1’s right elbow had a deterioration of (+3) point scores [score change of (+1) for surface erosions and (+2) for subchondral cysts]. There was a (+1) point score worsening in cartilage degradation in five index joints (four ankles and one elbow).

### 3.4. Standard Prophylaxis Group [Eight Boys Who Completed MRI Evaluations Were Available at Study Entry (48 Index Joints) and Exit (47 Index Joints)]

Similarly, in the SP group, the vast majority of index joints (41/47, 87.2%) did not show any changes reflected in MRI scores during the two-year study period. A change in the 17-point IPSG MRI score between study entry and exit of at least (±1) point score was observed in (6/47) 12.8% index joints in four of the eight (50%) boys randomized to receive standard prophylaxis [Table 3]. A score improvement of (−1) point was noted in the categories of effusion/hemarthrosis (left knee and left ankle, respectively), and synovial hypertrophy and hemosiderin (right ankle) in one participant. Worsening (+1 and +2) of effusion/hemarthrosis items was seen in the respective ankles of two boys. Concerning osteochondral subscores, a worsening of (+1) point score for cartilage degradation was observed in one participant’s ankle.

### 3.5. Correlations Among Scores of the Four MRI Scales and Between Scores of These Scales and Physical Examination and X-Ray Scales

Taking into account entry and exit data conjointly, moderate (upper end of category) to strong (lower end of category) correlations were noted between the 17-point IPSG scores and the two compatible scales (A-scale, r = 0.59, *p* = 0.009, borderline *p*-value at the Bonferroni-adjusted level of statistical significance; P-scale, r = 0.61, *p* = 0.007) and the Denver scale, r = 0.61, *p* = 0.007, in the TP arm [Table 4].

In the SP arm, very strong correlations were observed between total scores (sum of six index joints, taken at study entry and exit) of the 17-point IPSG scale, the compatible IPSG MRI-P scale (r = 0.95, *p* < 0.0001) and the Denver MRI scale (r = 0.95, *p* < 0.0001), and strong correlations between the 17-point IPSG scale and the compatible IPSG MRI-A scale (r = 0.65, *p* = 0.006) [Table 5].

It is of note that whereas the IPSG scores correlated strongly (r = 0.65, *p* = 0.006) with the compatible MRI A-scale in the SP arm, they correlated moderately (upper end, r = 0.59, *p* = 0.009, borderline *p*-value at the Bonferroni-adjusted level of statistical significance) with that scale in the TP arm [Table 4].

The two progressive scales (MRI-P and Denver scales) correlated strongly with the MRI A-scale in both study arms (TP: r = 0.67, *p* = 0.002 for MRI-P and r = r = 0.67, *p* = 0.002 for the Denver scale; and SP: r = 0.64, *p* = 0.008 for MRI-P and r = 0.64, *p* = 0.008 for the Denver scale). The Pettersson X-ray scale correlated strongly with the A- MRI scale (r = 0.78, *p* = 0.0002). Nevertheless, correlations with the 17-point IPSG MRI scale (r = 0.53, *p* = 0.02), the P- MRI scale (r = 0.57, *p* = 0.01) and the Denver MRI scale (r = 0.57, *p* = 0.01) in the TP arm, and with the 17-point IPSG MRI scale (r = 0.46, *p* = 0.07), the P- MRI scale (r = 0.48, *p* = 0.06) and the Denver MRI scale (r = 0.48, *p* = 0.06) in the SP arm did not achieve statistical significance at the adjusted alpha level of this study [Table 4 and Table 5].

The total scores of the Child PE scale did not correlate with any of the MRI or X-ray scales in any of the study arms [Table 4 and Table 5]. Correlation plots between total MRI scores for 17-point IPSG, compatible A- and P-, Denver MRI scales and Child PE total scores, considering the sum of six joints taken at study entry and exit, are provided in Supplementary Table S7.

Weak to moderate (lower end) correlations were noted when comparing the soft tissue total scores of the MRI scales (effusion/hemarthrosis, synovial hypertrophy and hemosiderin) to the swelling item of the Child PE scale [Supplementary Table S8].

Overall, considering both study arms conjointly, the inter-reader ICCs (95% CIs) for interpretation of MRI findings of the reference 17-point IPSG MRI scale were 0.95 (0.93–0.96) for total scores, 0.93 (0.91–0.95) for soft tissues and 0.95 (0.93–0.96) for osteochondral tissues. For individual items, they were, respectively, 0.83 (0.78–0.87) for effusion/hemarthrosis; 0.92 (0.90–0.94) for synovial hypertrophy; 0.89 (0.86–0.92) for hemosiderin; 0.86 (0.82–0.89) for surface erosions; 0.91 (0.88–0.93) for subchondral cysts and 0.89 (0.86–0.92) for cartilage loss. For the Pettersson C-ray scale, the total scores’ ICCs were 0.96 (0.93–0.98).

## 4. Discussion

**Figure 1:**
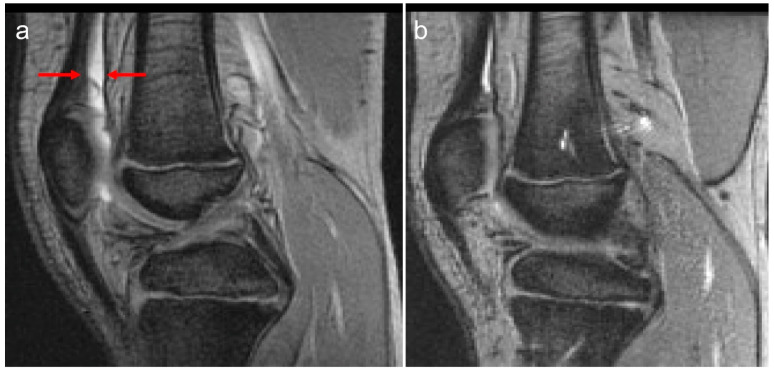
Ten-year-old boy (at baseline MRI) with severe hemophilia A, without prior left knee bleed history and no inhibitory antibodies. Baseline (**a**) and follow-up (**b**) MRI (**a**) examinations of the left knee show reversible soft tissue changes (effusion/hemarthrosis) of the knee. (**a**) The baseline sagittal multiplanar gradient-recalled (MRGR) MR image demonstrates an International Prophylaxis Study Group (IPSG) MRI score of 1: soft tissue domain = 1 (effusion/hemarthrosis = 1, arrows); osteochondral domain = 0. (**b**) The follow-up MR images fail to show any evidence of soft tissue or osteochondral tissue changes. Follow-up IPSG MRI score = 0; soft tissue domain = 0; osteochondral domain = 0. A physiological amount of fluid is seen within the left knee joint.

**Figure 2:**
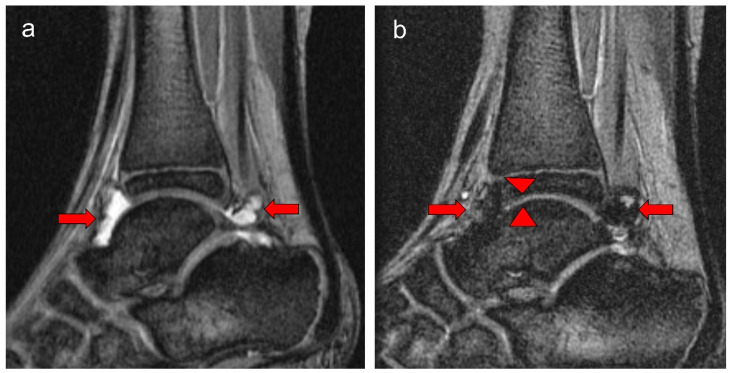
Eight-year-old boy (at baseline MRI) with severe hemophilia A, and history of 9 prior left ankle bleeds and no inhibitory antibodies. Baseline (**a**) and follow-up (**b**) MR images show reversible soft tissue changes (effusion/hemarthrosis) and irreversible osteochondral change (cartilage loss) of the ankle. (**a**) The baseline sagittal multiplanar gradient-recalled (MPGR) MR image of his ankle shows moderate effusion (short arrows), and mild hemosiderin deposition/synovial hypertrophy, International Prophylaxis Study Group (IPSG) MRI score = 4 (effusion/hemarthrosis = 2, synovial hypertrophy = 1, hemosiderin deposition = 1). (**b**) The corresponding follow-up MRI shows slight increase in hemosiderin deposition (arrows) compared with the baseline MRI superposed to minimal effusion. It also demonstrates minimal focal loss in cartilage height anteriorly at the tibiotalar joint level (arrowheads). IPSG MRI score = 8; soft tissue domain = 7; osteochondral domain = 1 (effusion/hemarthrosis = 1, synovial hypertrophy = 3, hemosiderin deposition = 3, cartilage loss = 1).

**Figure 3:**
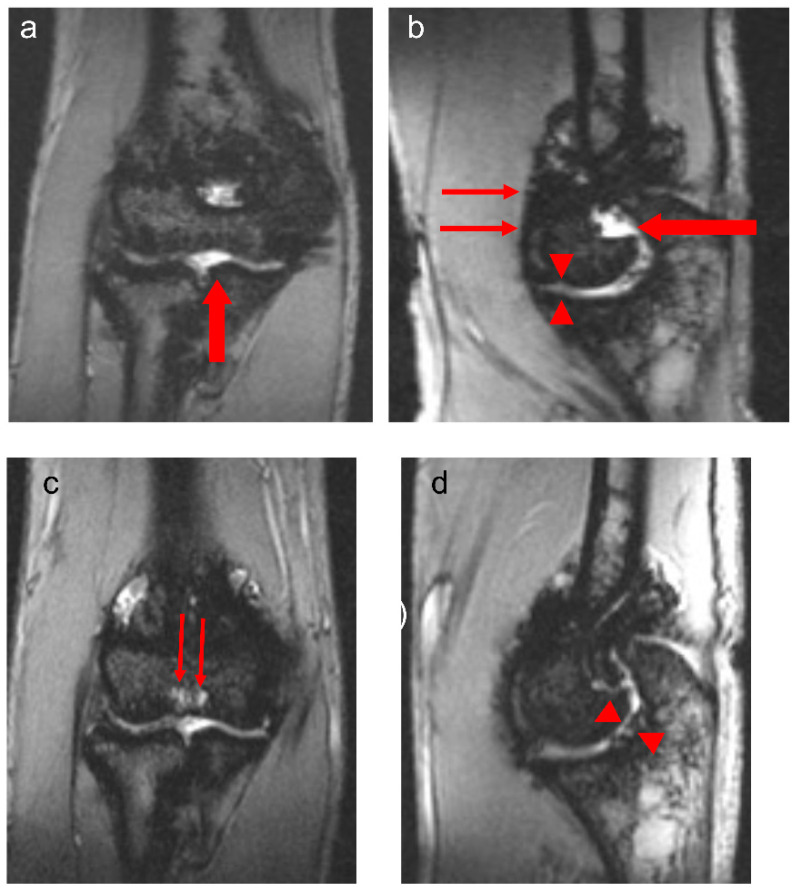
Thirteen-year-old boy with severe hemophilia A, with history of 1 prior right elbow bleed and no inhibitory antibodies. Baseline coronal (**a**) and sagittal (**b**) multiplanar gradient-recalled (MPGR) MR images of the right elbow show a small effusion/hemarthrosis (thick arrows) and superimposed marked synovial hypertrophy and hemosiderin deposition (thin arrows), and early erosive changes and cartilage loss (arrowheads, (**b**)). International Prophylaxis Study Group (IPSG) score = 9: soft tissue domain = 7 (effusion/hemarthrosis = 1, synovial hypertrophy = 3, hemosiderin deposition = 3); osteochondral domain = 2 (surface erosions = 1, cartilage loss = 1). Follow-up coronal (**c**) and sagittal (**d**) MPGR MR images show persistent joint cartilage loss associated with hemosiderin deposition (arrowheads, (**d**)). Subchondral cysts have developed in the distal humerus (thin arrows, (**c**)) in the interim. IPSG score = 10: soft tissue domain = 7 (effusion/hemarthrosis = 1, synovial hypertrophy = 3, hemosiderin deposition = 3); osteochondral domain = 3 (surface erosions = 1, subchondral cysts = 1, cartilage loss = 1).

The observations in this study are of importance in the context of the assessment of blood-related arthropathy in PwH. Given that MR imaging provides the greatest detail of blood-induced damage in the structures of joints in PwH, the overall relatively strong correlations noted between the total scores derived for the four MRI scales reported in the two study arms in this communication (Table 3) are reassuring. Whereas the Denver MRI scale has progressive methodological characteristics, i.e., only the worst change for each item is scored [12], the compatible MRI scales encompass progressive and additive methodological characteristics [13]. Nevertheless, for the additive component, i.e., each grading of the item is scored, synovial hypertrophy is semi-quantitatively scored (scores, 1–3), whereas hemosiderin deposition is assessed according to the presence (score 1) or absence (score 0) of the finding. The IPSG MRI scale, in contrast, provides the ability of detailed scoring (scores, 1–3) of the soft tissue joint components (effusion/hemarthrosis, synovial hypertrophy and hemosiderin deposition) [14] which in the case of this study results was important to demonstrate interval joint changes (Figure 1 and Figure 2). Furthermore, because of its additive capability of capturing osteochondral joint changes, it enables individualized assessment of joint changes over time concerning bone erosions, subchondral cysts and cartilage deterioration, presenting with separate items for bone erosions and subchondral cysts, conversely to the methodological design concept of the compatible MRI scales in which bone erosions and subchondral cysts are grouped into a single category. The characteristics of the osteochondral domain of the IPSG scale allowed discrimination of individual osteochondral changes in the joints of patients in this study (Figure 3). For the aforementioned reasons, particularly due to its ability to provide detailed information for both soft tissue and osteochondral changes and to give approximately equal weighting to both of these domains, the 17-point IPSG additive MRI scale was used as the reference scale for the results reported in this communication [14]. Similarly, several other publications in the literature have reported the use of the 17-point scale as a reference standard in their studies [21,22,23,24,25,26,27,28,29,30].

Progression (worsening) of MRI scores of the index joints in boys who received standard or tailored prophylaxis was observed both in the soft tissue and osteochondral domains (Table 3). Of the joints that deteriorated, eight were participants from the TP group while three were from the SP group. Further, the fact that 40.0% of participants of the TP arm and 25.0% of participants of the SP arm presented with deterioration of osteochondral structures of their joints supports the viewpoint that standard prophylaxis is not completely protective of bleed-related musculoskeletal damage in young boys with severe hemophilia A started on long-term prophylaxis from an early age in life [31]. Whether such changes, as detected by serial MRI studies, can be reversed by an intensification of the prophylaxis regimen in affected boys with severe hemophilia is a very important clinical question that deserves further study. In this study, we observed a correlation between joint swelling detected by physical examination of the index joints and the soft tissue total scores of all MRI scales. This is an important finding that deserves further study, as swelling reported on the Child PE clinically could relate to clinical and subclinical joint bleeding. If confirmed in boys with hemophilia and early joint disease, a combination of a physical examination of the index joints and ultrasound examination may identify joints that require a more detailed study with MRI. The resolution provided by MRI could then confirm or rule out early clinically significant hemophilic arthropathy and inform the appropriate change in prophylaxis regimen. This approach may be of value for soft tissue changes such as effusion, synovial hypertrophy and hemosiderin, but less relevant for established osteochondral changes. Nevertheless, the fact that the total scores of the Child PE scale did not correlate with any of the MRI or X-ray scales in any of the study arms is supported by similar results of other studies. A recent systematic review of measurement properties of hemophilia-specific instruments showed low correlations between the Colorado PE scale and the World Federation of Hemophilia (WFH) pain scale, and contradictory correlations with MRI scores [32]. In one pediatric study, correlations between Colorado PE scores and the additive and progressive MRI scores for elbows and knees were good, but unsatisfactory for ankles [23]. In another pediatric study, the overall Colorado PE scores did not correlate well with the overall MRI scores [33]. Currently, the tailored use of ultrasound, at point-of-care and full-joint basis, and MRI may provide valuable contributory information for physical examination findings, which can improve the sensitivity of diagnostic tools for subtle joint abnormalities in hemophilia [34]. Concerning full-joint ultrasound Doria et al. have shown that ultrasound was highly sensitive (>92%) for assessing synovial hypertrophy and hemosiderin (i.e., soft tissue changes) in both ankles and knees, but had variable sensitivity for evaluating osteochondral abnormalities (sensitivity range, 86–100% for ankles and 12–100% for knees) [25]. On the other hand, MRI has been considered the reference standard for the assessment of hemophilic joints in comparison with ultrasound [35], and could be integrated into clinical decision-making (e.g., treatment escalation), specifically when the assessment of subtle early osteochondral joint changes, not amenable to an accurate diagnosis by ultrasound, is needed.

One of the main limitations of this study was the relatively short interval of time, of approximately two years, between the MRI studies in boys who entered the study with mild/moderate arthropathy and were continued on prophylaxis. In the study conducted by Manco-Johnson et al. 65 boys with severe hemophilia A were followed from enrollment (before 30 months of age) until they reached 6 years old, with a follow-up period of approximately 3.5 to 4 years, depending on the age at enrollment [33]. The primary outcome was assessed when the boys reached 6 years of age. In the study conducted by Kraft et al. 24 boys with severe hemophilia A were followed for a median duration of approximately 7.2 years [23]. Subjects began therapy at a median age of 1.6 years (range: 1–2.5 years) and underwent MRI evaluations at a median age of 8.8 years (range: 6.2–11.5 years). In the study conducted by Stimec et al. 46 patients with severe hemophilia A were followed for a median duration of 9.6 years (range, 4.8 to 16.0 years) [30]. This extended follow-up period allowed for a comprehensive assessment of joint health outcomes using radiographs and MRI evaluations, allowing for to evaluation of the long-term effects of tailored primary prophylaxis on joint health in boys with severe hemophilia A.

Other limitations include the historical characteristics of this study, as recently the original IPSG MRI scale used for the data analysis of this study was updated to the IPSG MRI scale version 2.0 [Supplementary Table S9] [36], and the limited study sample size. Concerning the latter limitation, the small sample size of this study may have had an impact on the responsiveness to the change of the investigated MRI scales to interval changes at the two timepoints. Future studies with larger samples are needed to reassess the ability of specific MRI scales to detect minimal clinical differences in joint outcomes, thus supporting the results of this pilot study. Not surprisingly, because of the effectiveness of prophylaxis in preventing joint bleeds, interval changes in both soft tissue and osteochondral domains were small, most often of only 1 point, and in only 15.8% of the joints evaluated.

Note should be made that the updated IPSG MRI scale version 2.0 altered elements of the osteochondral domain only in relation to the original IPSG MRI scale, while maintaining all items of the soft tissue domain. In this study, the only notable interval change depicted by the IPSG MRI scale was a worsening of (+1) point score for cartilage degradation in one participant’s ankle. Therefore, we anticipate the minimal impact of the interval changes in the revised osteochondral domain of the IPSG MRI scale 2.0 on the results of this study.

Finally, for any observed change it is important to consider whether the change occurred in isolation or is part of a broader pattern of change in the same direction, as was noted in four boys in this study [Table 3, participants 1, 4, 9 of the TP arm and participant 16 of the SP arm]. It is also important to cross-reference serial changes in imaging studies to serial changes detected by a physical examination of the joint(s) in question by an experienced health care professional.

## 5. Conclusions

The strong and very strong correlations between the 17-point IPSG MRI scale and the progressive (MRI P- and Denver) MRI scales in the TP and SP arms of the study, respectively, and the borderline strong and strong correlations between the 17-point IPSG MRI scale and the A-MRI scale in the TP and SP arms of the study, respectively, confirm relative comparability of their abilities to measure what they propose to measure, i.e., construct validity, in boys with mild/moderate hemophilic arthropathy. This was reiterated by the fact that strong and moderate correlations were noted between the Pettersson X-ray scale and the A-, P-, Denver and 17-point IPSG MRI scales. However, only weak to moderate (lower end) correlations were noted when comparing the soft tissue total scores of the MRI scales to the swelling item of the Child PE scale, which, in addition to other physical examination measurement tools, deserve further investigation in the future. Given the high inter-reader correlation for soft tissue and osteochondral items of the 17-point IPSG MRI scale, it is recommended as the reference imaging standard for quantitation of mild/moderate arthropathy in boys with hemophilia. In subjects receiving effective long-term prophylaxis with clotting factor and non-factor-based hemostatic agents, the interval between serial MRIs will need to be longer than 2 years to allow detection of clinically significant osteochondral and soft tissue changes. To further confirm and validate the results of this preliminary study using refined MR imaging protocols, broader multicentric longitudinal studies with longer MRI intervals, beyond two years, are clearly needed.

## Figures and Tables

**Table 1:** Characteristics of study cohort at entry and exit.

	Standard Prophylaxis (SP) Study Arm Entry	Tailored Prophylaxis (TP) Study Arm Entry	*p* Value	Standard Prophylaxis (SP) Study Arm Exit	Tailored Prophylaxis (TP) Study Arm Exit	*p* Value
Number of participants	8	9		8	9	
Age (years)	10 (7–16) *	12 (8–15)	0.8290	–	–	
Child PE score	6 (2–15)	8 (3–16)	0.8943	6.5 (6–14)	13 (4–17)	0.6812
Pettersson X-ray score	0 (0–4)	2 (0–13)	0.5882	0 (0–4)	2 (0–14)	0.6985
	**MRI (17-point IPSG scale) scores for 6 index joints:**					
**Soft tissue total score**	**2.5 (0–11)**	**6 (0–15)**	**0.8137**	3 (0–7) **	**7 (1–14)**	**0.5798**
Effusion/hemarthrosis	0 (0–3)	2 (0–4)	0.1669	0.5 (0–2)	1 (0–3)	0.2519
Synovial hypertrophy	1 (0–3)	2 (0–6)	0.3973	1 (0–2)	2 (0–6)	0.2558
Hemosiderin	1 (0–5)	3 (0–6)	0.5579	1 (0–4)	3 (0–6)	0.4020
**Osteochondral total score**	**0 (0–4)**	**5 (0–10)**	**0.3240**	**0.5 (0–4)**	**6 (0–11)**	**0.3460**
Surface erosions	0 (0–1)	2 (0–3)	0.0005	0 (0–1)	2 (0–3)	0.0005
Subchondral cysts	0 (0–2)	1 (0–4)	0.1474	0 (0–2)	2 (0–4)	0.0429
Cartilage degradation	0 (0–2)	2 (0–4)	0.0429	0.5 (0–2)	2 (0–6)	0.1978
**Total score**	**5.5 (0–12)**	**11 (0–20)**	**0.7939**	**6 (0–8)**	**11 (1–22)**	**0.7046**

* Values are medians with ranges shown in parentheses. ** of 47 joints because one left elbow MRI was not performed due to patient’s lack of cooperation. Abbreviations: PE, physical examination.

**Table 2:** Frequency of joints of study cohort with MRI abnormalities as per the 17-point IPSG MRI scale.

	Standard Prophylaxis (SP) Study Arm Entry	Tailored Prophylaxis (TP) Study Arm Entry	*p*-Value	Standard Prophylaxis (SP) Study Arm Exit	Tailored Prophylaxis (TP) Study Arm Exit	*p*-Value
	**n (%)**	**n (%)**		**n (%)**	**n (%)**	
**Soft tissue items**	**12/48 (25.0)**	**19/54 (35.2)**	0.2887	11/47 (23.4) *	**20/54 (37.0)**	0.2751
Effusion/hemarthrosis	6/12	12/19	0.7098	6/11	11/20	0.7116
Synovial hypertrophy	6/12	9/19	1.0	6/11	9/20	1.0
Hemosiderin	9/12	13/19	1.0	9/11	14/20	0.6722
**Osteochondral items**	**3/48 (6.3)**	**11/54 (20.4)**	**0.0466**	4/47 (8.5) *	**13/54 (24.1)**	0.1593
Surface erosions	2/3	10/11	0.3956	2/4	10/13	0.1538
Subchondral cysts	2/3	7/11	1.0	2/4	8/13	1.0
Cartilage degradation	3/3	11/11	1.0	4/4	13/13	1.0
**Total items**	**12/48 (25.0)**	**23/54 (42.6)**	0.0940	11/47 (23.4) *	**23/54 (42.6)**	**0.0574**

* of 47 joints because one left elbow MRI was not performed due to patient’s lack of cooperation. n = number.

**Table 3:** Joint and item scores by participant, at study entry and exit where there was a change in score ≥ 1 using the 17-point IPSG MRI scale.

Arm	Participant	Index Joint	Joint Entry Score	Joint Exit Score	IPSG 17-Point Item Element that Changed	Item Entry Score	Item Exit Score	Total Change by Item
Tailored Prophylaxis	1	RE	9	12	surface erosions	1	2	1
					subchondral cysts	0	2	2
		LA	1	0	effusion/hemarthrosis	1	0	−1
	2	LA	4	8	effusion/hemarthrosis	2	1	−1
					synovial hypertrophy	1	3	2
					hemosiderin	1	3	2
					cartilage degradation	0	1	1
	3	LA	5	6	hemosiderin	0	1	1
	4	LA	4	5	cartilage degradation	0	1	1
		RA	7	6	effusion/hemarthrosis	2	0	−2
					cartilage degradation	1	2	1
	5	LA	0	1	effusion/hemarthrosis	0	1	1
	6	RA	9	10	hemosiderin	1	2	1
	9	LE	3	4	cartilage degradation	1	2	1
		LA	3	4	cartilage degradation	1	2	1
Standard Prophylaxis	16	LK	1	0	effusion/hemarthrosis	1	0	−1
		LA	1	0	effusion/hemarthrosis	1	0	−1
		RA	8	6	synovial hypertrophy	3	2	−1
					hemosiderin	3	2	−1
	17	LA	4	5	cartilage degradation	0	1	1
	18	RA	6	7	effusion/hemarthrosis	0	1	1
	20	LA	0	2	effusion/hemarthrosis	0	2	2

Abbreviations: index joints are defined as left (L) or right (R) ankle (A) and elbow (E) joints. Note: improvement in scores was reported as −1, −2, −3, where −3 had the most improvement in findings, stability as 0, and worsening as +1, +2, +3, where +3 had the most disease progression.

**Table 4:** Paired Spearman correlations (*p*-values) of total scores of the four MRI scales and the Child Physical Examination (PE) scale (sum of six index joints taken at study entry and exit). Tailored arm (entry and exit conjointly, N = 18).

Values	MRI Scales	17-Point IPSG Scale	MRI A-Scale	MRI P-Scale	Denver Scale	Child PE Scale	Pettersson
r-value	17-point IPSG scale	**1.00**	* 0.59	**0.61**	**0.61**	0.02	0.53
*p*-value		**<0.0001**	**0.009**	**0.007**	**0.007**	0.93	0.02
r-value	MRI A-scale	* 0.59	**1.00**	**0.67**	**0.67**	0.51	**0.78**
*p*-value		**0.009**	**<0.0001**	**0.002**	**0.002**	0.03	**0.0002**
r-value	MRI P-scale	**0.61**	0.67	**1.00**	**1.00**	0.37	0.57
*p*-value		**0.007**	0.002	**<0.0001**	**<0.0001**	0.13	0.01
r-value	Denver scale	**0.61**	**0.67**	**1.00**	**1.00**	0.37	0.57
*p*-value		**0.007**	**0.002**	**<0.0001**	**<0.0001**	0.13	0.01
r-value	Child PE scale	0.02	0.51	0.37	0.37	**1.00**	0.28
*p*-value		0.93	0.03	0.13	0.13	**<0.0001**	0.25
r-value	Pettersson	0.53	**0.78**	0.57	0.57	0.28	**1.00**
*p*-value		0.02	**0.0002**	0.01	0.01	0.25	**<0.0001**

Bonferroni-adjusted *p*-values (threshold for statistical significance, p < 0.008 at the adjusted level). * Borderline statistical level of significance. Results in bold are statistically significant.

**Table 5:** Paired Spearman correlations (*p*-values) of total scores of the four MRI scales and the Child Physical Examination (PE) scale (sum of six index joints taken at study entry and exit). Standard arm (entry and exit conjointly, N = 16).

Values	MRI Scales	17-Point IPSG Scale	MRI A-Scale	MRI P-Scale	Denver Scale	Child PE Scale	Pettersson
r-value	17-point IPSG scale	**1.00**	**0.65**	**0.95**	**0.95**	−0.16	0.45
*p*-value		**<0.0001**	**0.006**	**<0.0001**	**<0.0001**	0.55	0.07
r-value	MRI A-scale	**0.65**	**1.00**	* 0.64	* 0.64	0.016	−0.22
*p*-value		**0.006**	**<0.0001**	**0.008**	**0.008**	0.95	0.41
r-value	MRI P-scale	**0.95**	* 0.64	**1.00**	**1.00**	−0.18	0.48
*p*-value		**<0.0001**	**0.008**	**<0.0001**	**<0.0001**	0.50	0.06
r-value	Denver scale	**0.95**	* 0.64	**1.00**	**1.00**	−0.18	0.48
*p*-value		**<0.0001**	**0.008**	**<0.0001**	**<0.0001**	0.50	0.06
r-value	Child PE scale	−0.16	0.02	−0.18	−0.18	**1.00**	−0.21
*p*-value		0.55	0.9	0.50	0.50	**<0.0001**	0.44
r-value	Pettersson	0.46	−0.22	0.48	0.48	−0.21	1.00
*p*-value		0.07	0.41	0.06	0.06	0.44	**<0.0001**

Bonferroni-adjusted *p*-values (threshold for statistical significance, *p* < 0.008 at the adjusted level). * Borderline statistical level of significance. Abbreviations: IPSG, International Prophylaxis Study Group; A, additive; P, progressive; PE, physical examination. Results in bold are statistically significant.

## Data Availability

All data generated or analyzed during this study are included in this published article [and its Supplementary Information Files].

## Supplementary Materials

The following supporting information can be downloaded at: https://www.mdpi.com/article/10.3390/jcm14134792/s1, Table S1: Colorado Child Physical Examination [9,10]; Table S2: Pettersson X-Ray Scale [11]; Table S3: Denver MRI Scale [12]; Table S4: Compatible MRI Scoring System (IPSG P-scale and A-scale) [13]; Table S5: International Prophylaxis Study Group (IPSG) 17-Point MRI Scale [14]; Table S6: Frequency of joints with abnormalities (score ≥ 1) (percentage in relation to number of joints evaluated) detected by the 17-point International Prophylaxis Study Group (IPSG) in ankles, elbows and knees of study patients at study entry or exit in the tailored and standard prophylaxis groups combined (n = 17); Table S7: Correlation plots of paired Spearman correlations: total scores of MRI scales and Child Physical Examination scale (sum of 6 joints taken at study entry and exit); Table S8. Paired Spearman correlations of soft tissue total scores of MRI scales and swelling item of Child Physical Examination scale (sum of 6 joints taken at study entry and exit); Table S9: Revised International Prophylaxis Study Group (IPSG) 17-Point MRI Scale 2.0 [36].
