# Supplementary material for: Comparison of Magnetic Resonance Imaging Scales for Assessment of Interval Changes of Arthropathy in Boys with Severe Hemophilia
Source: J Clin Med. 2025 Jul 7;14(13):4792. doi: 10.3390/jcm14134792 (PMC12251000; doi:10.3390/jcm14134792)
Supplement: Supplementary file 1 [file jcm-14-04792-s001.zip › jcm-3635606-supplementary.pdf]

Supplementary Material

Supplementary Table S1. Colorado Child Physical Examination [9, 10]

Physical finding	Score	Scoring key
**Swelling**	0–3	0 = none<br>1 = joint looks slightly 'puffy'; there is slight palpable swelling present; may not be any measurable difference between the joints; bony landmarks clearly visible.<br>2 = joint looks swollen and the swollen area feels firm on palpation; may also be feel boggy; there is measurable difference between the joints; bony landmarks are palpable but not visible<br>3 = swollen and are tense to palpation; there is measurable difference between the joints and the bony landmarks are difficult to palpate.
**Muscle atrophy**	0–3	0 = none<br>1 = muscle has slightly less contour than the contralateral side.<br>2 = flattening of the muscle belly.<br>3 = severe muscle wasting and depression.
Axial deformity		
Knee:	0–2	0 = normal; 0–7° valgus<br>1 = 8–15° valgus or 0–5° varus<br>2 = > 15° valgus or > 5° varus
Ankle:	0–2	0 = no deformity<br>1 = up to 10° valgus or 1–5° varus<br>2 = > 10° valgus, or > 5° varus
**Crepitus with motion**	0–3	0 = none<br>1 = barely detectable audible or palpable sensation during joint motion<br>2 = more pronounced cracking and/or rough sensation during joint motion<br>3 = audible and palpable grinding and crunching during joint motion
**Range of motion**	0–3	0 = no loss<br>1 = loss of < 10% of total FROM<br>2 = loss of 10–33% of total FROM<br>3 = loss of > 33% of total FROM
Flexion contracture: measured at hip, knee, ankle and elbow	0–3	0 = normal<br>1 = 0–7°<br>2 = 8–15°<br>3 = > 15°
Instability		
**New additions:**		Deleted
**Pain with activity**	0–3	Uses Faces Pain Rating Scale (Wong–Baker)<br>0 = Face is very happy<br>1 = Wong–Baker faces 1 & 2: hurts a little bit or a little bit more<br>2 = Wong–Baker face 3: hurts even more<br>3 = Wong–Baker faces 4 & 5: hurts a whole lot and as much as you can imagine
**Pain without activity**	0–3	Uses Faces Pain Rating Scale (Wong–Baker)<br>0 = Face is very happy<br>1 = Wong–Baker faces 1 & 2: hurts a little bit or a little bit more<br>2 = Wong–Baker face 3: hurts even more<br>3 = Wong–Baker faces 4 & 5: hurts a whole lot and as much as you can imagine
**Gait**	0–3	0 = normal walking, running, skipping, galloping, stairs<br>1 = normal walking, one or more other gait abnormality<br>2 = abnormal walking and ≤2 other gait abnormalities<br>3 = abnormal walking and > 2 gait abnormalities
**Strength**	0–3	0 = moves easily through full ROM against gravity without observable/measurable atrophy and can take additional resistance<br>1 = moves through available ROM, easily against gravity, may have observable/measurable atrophy and can take some additional muscle resistance<br>2 = moves through full or available ROM against gravity, cannot take resistance<br>3 = unable to move through full or available ROM against gravity due to weakness
**Splinting/Orthotics**	0–3	0 = no use of splinting/orthotics<br>1 = splinting/orthotic use required as needed after an acute haemarthrosis or for occasional support<br>2 = splinting/orthotic use required regularly for high activity sports or to prevent recurrent haemarthrosis<br>3 = splinting/orthotic use required continuously
Total	0–31	Ankle or Knee
	0–29	Elbow

Supplementary Table S2. Pettersson X-Ray Scale [11]

**Findings**		**Score**
Osteoporosis	Absent	0
	Present	1
Enlarged Epiphysis	Absent	0
	Present	1
Irregular Subchondral Surface	Absent	0
	Partially involved	1
	Totally involved	2
Narrowing of Joint Spaces	Absent	0
	Visible joint space >1mm	1
	Visible joint space <1mm	2
Subchondral Cyst Formation	Absent	0
	1 cyst	1
	>1 cyst	2
Erosion of Joint Margins	Absent	0
	Present	1
Gross Incongruence of Articulating Bone Ends	Absent	0
	Slight	1
	Pronounced	2
Joint Deformity (Angulation and/or Displacement)	Absent	0
	Slight	1
	Pronounced	2
**Total Score (sum all points /13)**		

Supplementary Table S3. Denver MRI Scale [12]

Commented [AD1]: Correct reference is #12, not #10.

DENVER MRI SCORE								
FINDINGS		Ankle		Knee		Elbow	
		L	R	L	R	L	R
Effusion	Absent	0	0	0	0	0	0
	Small	1	1	1	1	1	1
	Moderate	2	2	2	2	2	2
	Large	3	3	3	3	3	3
Hemarthrosis	Absent	0	0	0	0	0	0
	Small	1	1	1	1	1	1
	Moderate	2	2	2	2	2	2
	Large	3	3	3	3	3	3
Synovial Hyperplasia	Absent	0	0	0	0	0	0
	Small	4	4	4	4	4	4
	Moderate	5	5	5	5	5	5
	Large	6	6	6	6	6	6
Hemosiderin	Absent	0	0	0	0	0	0
	Small	4	4	4	4	4	4
	Moderate	5	5	5	5	5	5
	Large	6	6	6	6	6	6
Erosion	Absent	0	0	0	0	0	0
	Partial surface erosion	7	7	7	7	7	7
	Full surface erosion	8	8	8	8	8	8
Subchondral Cyst	Absent	0	0	0	0	0	0
	1 cyst	7	7	7	7	7	7
	> 1 cyst	8	8	8	8	8	8
Cartilage Loss	Absent	0	0	0	0	0	0
	Less than 50% loss	9	9	9	9	9	9
	50% or greater loss	10	10	10	10	10	10
**MRI SCORE** (highest number in any category)							

Supplementary Table S4. Compatible MRI Scoring System (IPSG P-scale and A-scale) [13]

Commented [AD2]: Correct reference is #13, not #11.

	Progressing scale (p)	Additive scale (a)
**Effusion/hemarthrosis**		
Small	(1)	
Moderate	(2)	
Large	(3)	
**Synovial Hypertrophy**		
Small	(4)	(1)
Moderate	(5)	(2)
Large	(6)	(3)
**Hemosiderin**		
Present		(1)
Small	(4)	
Moderate	(5)	
Large	(6)	
**Changes of subchondral bone or joint margins**		
Any surface erosion	(7)	(1)
Any surface erosion in at least 2 bones		(1)
Half or more of the articular surface eroded in at least one bone	(8)	(1)
Half or more of the articular surface eroded in at least two bones		(1)
At least one subchondral cyst	(7)	(1)
More than one subchondral cyst	(8)	(1)
Subchondral cysts in at least two bones		(1)
Multiple subchondral cysts in each of at least two bones		(1)
**Cartilage loss**		
Any loss of joint cartilage height	(9)	(1)
Any loss of joint cartilage height in at least two bones		(1)
Any loss of joint cartilage height involving more than one third of joint surface in at least one bone		(1)
Any loss of joint cartilage height involving more than one third of joint surface in at least two bones		(1)
Full-thickness loss of joint cartilage in at least some area in at least one bone	(10)	(1)
Full-thickness loss of joint cartilage in at least some area in at least two bones		(1)
Full-thickness loss of joint cartilage involves at least one third of joint surface in at least one bone		(1)
full-thickness loss of joint cartilage involves at least one third of joint surface in at least two bones		(1)
	Record only the highest number (max value = 10)	Add numbers (max value = 20)
**SCORES**	**(P)**	**(A)**

Supplementary Table S5. International Prophylaxis Study Group (IPSG) 17-Point MRI Scale [14]

Commented [AD3]: Correct reference is #14, not #12.

SOFT TISSUE		
Effusion/Hemarthrosis	small	(1)________
	moderate	(2)________
	Large	(3)________
Synovial Hypertrophy	small	(1)________
	moderate	(2)________
	Large	(3)________
Hemosiderin	small	(1)________
	moderate	(2)________
	Large	(3)________
Soft tissue changes subscore	*Maximum 9*	________
**OSTEOCHONDRAL**		
Surface Erosions involving Subchondral Cortex or Joint Margins	any surface erosion	(1)___
	half or more of the articular surface eroded in at least one bone	(1)___
Subchondral Cysts	at least one subchondral cyst	(1)___
	subchondral cysts in at least two bones, or cystic changes involving a third or more of the articular surface in at least one bone	(1)___
Cartilage Degradation	any loss of joint cartilage height	(1)___
	loss of half or more of the total volume of joint cartilage in at least one bone	(1)___
	full-thickness loss of joint cartilage in at least some area of at least one bone	(1)___
	full-thickness loss of joint cartilage including at least one half of the joint surface in at least one bone	(1)___
Osteochondral changes subscore	*Maximum 8*	_____
**SCORING TOTAL**	**Maximum 17 (9/8)**	_____

Supplementary Table S6. Frequency of joints with abnormalities (score ≥ 1) (percentage in relation to number of joints evaluated) detected by the 17-point International Prophylaxis Study Group (IPSG) in ankles, elbows and knees of study patients at study entry or exit in the tailored and standard prophylaxis groups combined (n=17)

Item	Right ankle		Left ankle		Right elbow		Left elbow		Right knee		Left knee	
	Entry (%)	Exit (%)	Entry (%)	Exit (%)	Entry (%)	Exit (%)	Entry (%)	Exit* (%)	Entry (%)	Exit (%)	Entry (%)	Exit (%)
**Soft Tissue**												
Effusion/hemarthrosis	5 (29.4)	5 (29.4)	6 (35.3)	6 (35.3)	2 (11.8)	2 (11.8)	2 (11.8)	2 (12.5)	0 (0.0)	0 (0.0)	1 (5.9)	1 (5.9)
Synovial hypertrophy	7 (41.2)	7 (41.2)	4 (23.5)	4 (23.5)	2 (11.8)	2 (11.8)	2 (11.8)	2 (12.5)	0 (0.0)	0 (0.0)	0 (0.0)	0 (0.0)
Hemosiderin	7 (41.2)	7 (41.2)	5 (29.4)	6 (35.3)	5 (29.4)	5 (29.4)	5 (29.4)	5 (31.3)	0 (0.0)	0 (0.0)	0 (0.0)	0 (0.0)
**Osteochondral**												
Surface erosions	4 (23.5)	4 (23.5)	2 (11.8)	2 (11.8)	2 (11.8)	2 (11.8)	3 (17.6)	3 (18.8)	0 (0.0)	0 (0.0)	1 (5.9)	1 (5.9)
Subchondral cysts	4 (23.5)	4 (23.5)	2 (11.8)	2 (11.8)	1 (5.9)	2 (11.8)	2 (11.8)	2 (12.5)	0 (0.0)	0 (0.0)	0 (0.0)	0 (0.0)
Cartilage loss	6 (35.3)	6 (35.3)	2 (11.8)	4 (23.5)	2 (11.8)	2 (11.8)	3 (17.6)	3 (18.8)	0 (0.0)	0 (0.0)	1 (5.9)	1 (5.9)

*(n=16) for left elbow exit MRI

Supplementary Table S7. Correlation plots of paired Spearman correlations: total scores of MRI scales and Child Physical Examination scale (sum of 6 joints taken at study entry and exit).

Commented [AD4]: Correct: plots, not plot.

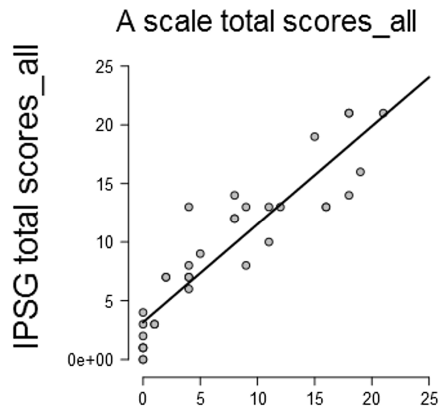

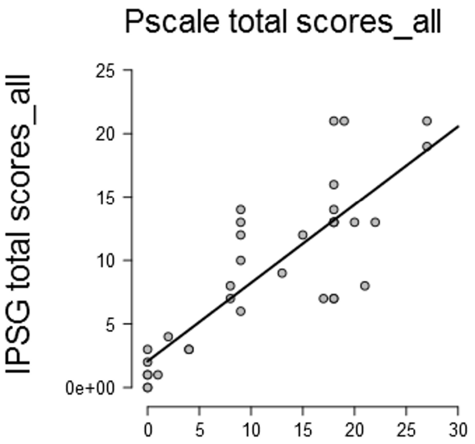

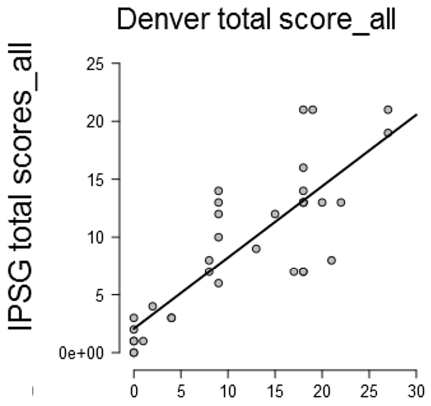
Denver total score_all
IPSG total scores_all
25
20
15
10
5
0e+00
0
5
10
15
20
25
30

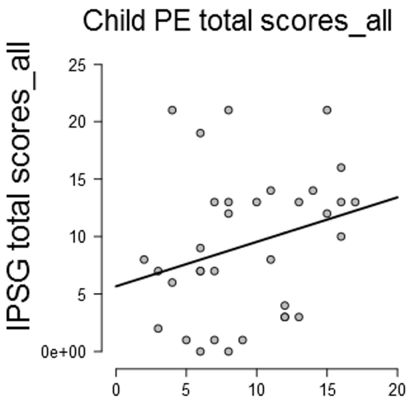
Child PE total scores_all
IPSG total scores_all
25
20
15
10
5
0e+00
0
5
10
15
20

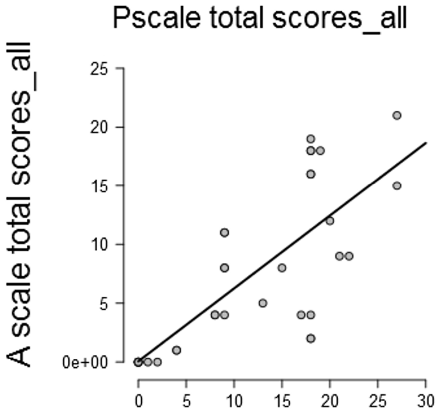
Pscale total scores_all
A scale total scores_all
25
20
15
10
5
0e+00
0
5
10
15
20
25
30

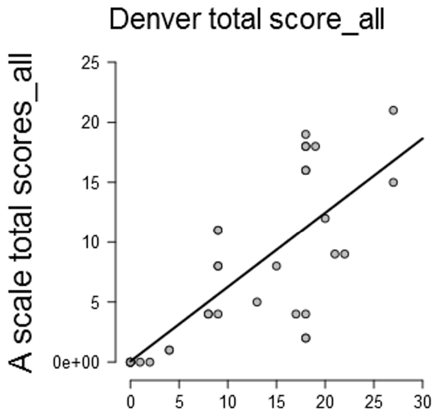
Denver total score_all
A scale total scores_all
25
20
15
10
5
0e+00
0
5
10
15
20
25
30

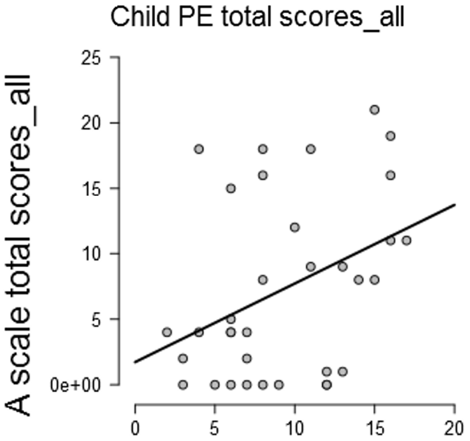
Child PE total scores_all
A scale total scores_all
25
20
15
10
5
0e+00
0
5
10
15
20

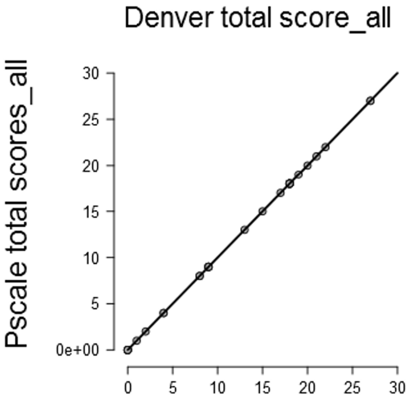
Denver total score_all
Pscale total scores_all
30
25
20
15
10
5
0e+00
0
5
10
15
20
25
30

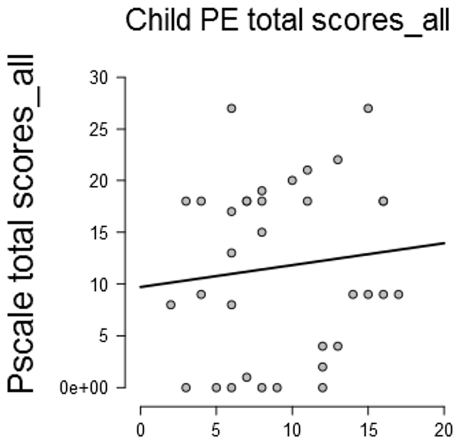
Child PE total scores_all
Pscale total scores_all
30
25
20
15
10
5
0e+00
0
5
10
15
20

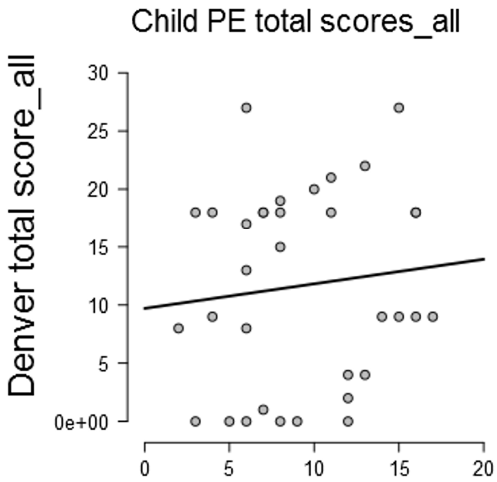
Child PE total scores_all
Denver total score_all
30
25
20
15
10
5
0e+00
0
5
10
15
20

Supplementary Table S8. Paired Spearman correlations of soft tissue total scores of MRI scales and swelling item of Child Physical Examination scale (sum of 6 joints taken at study entry and exit)

			Spearman's rho		p	Lower 95% CI	Upper 95% CI
IPSG soft tissue total	-	P scale soft tissue total	0.807	***	< .001	0.644	0.900
IPSG soft tissue total	-	A scale soft tissue total	0.861	***	< .001	0.737	0.929
IPSG soft tissue total	-	Denver soft tissue total	0.807	***	< .001	0.644	0.900
IPSG soft tissue total	-	Child PE swelling	0.433	*	0.011	0.111	0.673
P scale soft tissue total	-	A scale soft tissue total	0.903	***	< .001	0.814	0.951
P scale soft tissue total	-	Denver soft tissue total	1.000	***	< .001	1.000	1.000
P scale soft tissue total	-	Child PE swelling	0.350	*	0.043	0.013	0.615
A scale soft tissue total	-	Denver soft tissue total	0.903	***	< .001	0.814	0.951
A scale soft tissue total	-	Child PE swelling	0.278		0.112	-0.067	0.563
Denver soft tissue total	-	Child PE swelling	0.350	*	0.043	0.013	0.615

* p < .05, ** p < .01, *** p < .001

Abbreviations: CI, confidence interval; PE, physical examination

Supplementary Table S9. Revised International Prophylaxis Study Group (IPSG) 17-Point MRI Scale 2.0 [38]

Commented [AD5]: Correct reference is #14, not #12.

SOFT TISSUE CHANGES

Effusion/Haemarthrosis
Small (one point)
Moderate (two points)
Large (three points)

Synovial hypertrophy
Small (one point)
Moderate (two points)
Large (three points)

Haemosiderin
Small (one point)
Moderate (two points)
Large (three points)

Soft tissue domain subscore

Maximum value 9

OSTEOCHONDRAL CHANGES

(Note: when scoring these items each positive finding adds one point
Which makes maximal values two points for bone erosions and/or subchondral endplate irregularity, two points for subchondral cysts and four points for cartilage degradation)

Bone erosions and/or subchondral endplate irregularities

Any surface erosion (one point)
Any bone erosion involving the joint margins, or irregularity of the endplate, in any bone (one point)
Irregularity of >50% of the endplate area in any of the two largest bones (one point)

Subchondral cysts
Subchondral cyst(s) in any bone (one point
Subchondral cysts(s) in >50% of the subarticular bone in any of the two largest bones (one point)

Cartilage degradation
Any loss of cartilage height in >50% of the articular surface area in any of the two largest bones (one point)
Loss of >50% of the cartilage height anywhere in any of the two largest bones (one point)
Loss of >50% of the cartilage height in >50% of the articular surface area in any of the two largest bones (one point)

Osteochondral domain subscore
Maximum value 8

Maximum total score 17 (9/8)
